# *Polygonum cuspidatum* Loaded Nanostructured Lipid Carriers for Dual Inhibition of TNF-α- and IL-6 Cytokines and Free Radical Species

**DOI:** 10.3390/ma16093492

**Published:** 2023-05-01

**Authors:** Teodora-Alexandra Iordache, Nicoleta Badea, Mirela Mihaila, Simona Crisan, Anca Lucia Pop, Ioana Lacatusu

**Affiliations:** 1 Faculty of Chemical Engineering and Biotechnologies, University Politehnica of Bucharest, Polizu No. 1, 011061 Bucharest, Romania; teodora.manasia@stud.chimie.upb.ro (T.-A.I.); nicoleta.badea@upb.ro (N.B.); 2 National Research & Development Institute for Food Bioresources—IBA Bucharest, 6th Dinu Vintila Street, 021101 Bucharest, Romania; 3 Stefan S. Nicolau Institute of Virology, Mihai Bravu Street No. 285, 030304 Bucharest, Romania; mirela.mihaila@virology.ro; 4 Faculty of Pharmacy, Titu Maiorescu University, 040314 Bucharest, Romania; 5 R.D. Center, A.C. HELCOR, Victor Babes Street, 430082 Baia Mare, Romania; simonacrisan@achelcor.ro; 6 Faculty of Pharmacy, “Carol Davila” University of Medicine and Pharmacy, 6 Traian Vuia Street, 020945 Bucharest, Romania

**Keywords:** phytochemical active principle, lipid delivery systems, free radical species, long-live cationic radicals, pro-inflammatory cytokines inhibition

## Abstract

The main objective of this study was the testing of natural compounds, such as *Polygonum cuspidatum* (PgnC) loaded into nanostructured lipid carriers (NLC), which can act as a “double-edged sword” aimed at simultaneously combating dangerous free radicals and inhibiting pro-inflammatory cytokines. Resveratrol-rich PgnC extract was paired with another phytochemical, Diosgenin (DSG), in NLC. The lipid nanocarriers carrying both herbals (NLC-DSG-PgnC) had spherical diameters (100 ± 2 50 nm), a polydispersity index of ~0.15, and electrokinetic potentials greater than −46.5 mV. Entrapment efficiencies of 65% for PgnC and 87% for DSG were determined by chromatographic and UV-Vis spectroscopy assays. Cell cytotoxicity analysis proved that 50 µg/mL of NLC-PgnC and dual-NLC ensured a biocompatible effect like the untreated cells. The dual-NLC assured a much slower in vitro release of DSG and PgnC (67% PgnC and 48% DSG) than the individual-NLC (78% PgnC and 47% DSG) after 4 h of experiments. NLC encapsulating PgnC presented a superior ability to capture cationic radicals: 74.5 and 77.9%. The chemiluminescence results pointed out the non-involvement of DSG in stopping oxygenated free radicals, while the antioxidant activity was maintained at a level higher than 97% for dual-NLC. NLC-DSG-PgnC ensured a promising capacity for inhibition of pro-inflammatory cytokine IL-6, ranging from 91.9 to 94.9%.

## 1. Introduction

Plant extracts, rich in phytochemicals, have attracted scientists’ attention through the multitude of benefits they provide with minimal or lack of side effects [1]. Following the natural-based products trend, consumers prefer them, so it is necessary to develop and apply fast and reliable technologies to support the great demands of the growing population. According to the World Health Organization, the demand for herbal products increased to almost 50% in developed states, especially after the pandemic crisis, not to mention that in underdeveloped countries they are the only available choice [2,3].

Since ancient times, people have used medicinal plants to prevent/treat several medical conditions. Still, the use of natural products is not easy, as it raises many challenges associated with the wide range of unique plant features (biological, chemical, and species-specific) [4]. Among these challenges, the most difficult ones to overcome have been limited bioavailability in aqueous medium, poor permeability over the gastrointestinal tract, and lack of a specific target. In this respect, nanotechnology plays a vital role through the development of novel delivery systems tailored to exceed these deficiencies [5,6]. During the recent decades, polymeric, metallic, magnetic, and lipid nanoparticles have been a focal point for scientists endeavoring to overcome the above-mentioned drawbacks. Nanostructured lipid carriers (NLCs), owing to their compatibility with the human body and their ability to increase drug solubility and improve herbal extracts’ traits in order to maximize their therapeutic outcomes, stood out as a perfect choice [5,7]. NLCs are present in the literature of recent years as efficient solid drug delivery systems with the previously listed advantages [8,9]. They consist of a lipid core composed of GRAS (generally recognized as safe) lipids stabilized by biocompatible surfactants and co-surfactant(s) [10]. Unlike the previous version of lipid nanoparticles, solid lipid nanoparticles (SLN), the lipid matrix of NLCs contain a blend of liquid lipids and solid lipids. This combination allowed for the alteration of the perfect crystalline arrangement, enabling a higher amount of extract to be integrated [11]. Moreover, NLC has some additional advantages, such as increased physical stability, high entrapment efficiency for different types of extracts, prolonged controlled release, and lighter implementation on a larger scale [12]. These lipid nanocarriers have already been used in experimental trials encapsulating different types of herbal extracts (e.g., glycyrrhiza glabra extract-based lipid nanocarriers, which showed appropriate antioxidant and anti-inflammatory actions [13]; carrot extract, marigold extract, and willow bark extract NLC, which demonstrated improved elasticity, hydration effect and antioxidant action [14,15]). All of them proved to have good efficacy, but still, there are requirements for continuous progression in active phytochemicals’ valorization for pharmaceutical purposes. In light of the need for a deeper view of the therapeutic benefits and enhancement of the synergism between the active selected extracts, Polygonum cuspidatum (PgnC) standardized in 50% Resveratrol and Diosgenin (DSG) was defined as the target of the present study.

*Polygonum cuspidatum* (PgnC), belonging to Polygonum family, is a perennial plant mostly spread on the Asian continent. Since ancient times, the root of this plant has served medicinal purposes in treating atherosclerosis, cough, asthma, hypertension, and cancer [16,17]. The positive aspects are related to the active constituents, notably resveratrol, polydatin, physcion, anthraglycoside B, emodin, apigenin, rhein, catechin, quercetin, and coumarins [18]. The most abundant of these is resveratrol. Resveratrol (3,5,4′-trihydroxy-stilbene) is a polyphenolic phytoconstituent, part of the stilbene family, known for its broad range of healing actions (anti-carcinogenic, anti-inflammatory, anti-obesity, antioxidant, and cardioprotective) [19]. However, its availability is limited given its poor water solubility, low bioavailability, and the chemical instability which it can go through, especially along the gastrointestinal tract. Resveratrol’s bioavailability is less than 1% due to extensive metabolism in the intestine and liver; it suffers sulfation and glucuronic reactions with generation of metabolites [20]. Consequently, only trace concentrations of free resveratrol can be found in the systemic circulation. For this reason, in order to make the best of resveratrol’s advantages, encapsulation proved to be the fastest, most trustworthy, and most sustainable option. Synthesized polymeric particles loading resveratrol (e.g., bovine serum albumin-based nanoparticles (NPs) [21], polylactic-co-glycolic acid [PLGA]-oil hybrid NPs [22]) helped to increase the antioxidant activity and raise the biocompatibility, stability, or targeted release. Different types of liposomes (PEG-modified liposomes containing resveratrol, succinyl chitosan-stabilized liposomes, peptide cationic liposomal nanocarrier, alginate–sucrose, and alginate–chitosan microbeads containing liposomes) have upgraded the favorable in vivo properties of resveratrol [23]. Pharmacokinetics difficulties, as previously mentioned, were surpassed by β-cyclodextrins encapsulation [17,24]. Resveratrol loaded in micelles was proven to be effective against oxidative stress, to improve controlled release, and to have greater exposure time in blood circulation, favoring reduction of breast cancer cells [17,25]. Lipid nanoparticles have also served as delivery systems for resveratrol (SLN and NLC). Miao Liu et al. prepared resveratrol-loaded-NLC hydrogel in order to improve stability and resveratrol absorption at the skin surface and to fight UV radiation repercussions [26]. The same systems were earlier used by Neves et al. in the pursuit of resveratrol protection via the gastrointestinal tract (GIT) and better permeability through the intestinal barrier [27].

The same shortcomings and deficiencies associated with low bioavailability and stability in different circumstances have been reported for Diosgenin (DSG), a steroidal sapogenin, which occurs abundantly in plants such as *Dioscorea alata*, *Smilax china*, and *Trigonella foenum graecum* [28]. This phytochemically active principle is currently being used as an important raw material for the preparation of several steroidal drugs in the pharmaceutical industry. Still, it has garnered high interest for the treatment of disorders such as hypercholesterolemia, inflammation, and several types of infections [29,30]. Different types of biocompatible lipid coatings were developed in order to overcome the above-mentioned limitations, and our group established a connection between DSG and glycyrrhiza glabra to enhance the antioxidant potential [13,30]. Related research investigated the anticancer and antidepressant effect of stearic acid solid lipid nanoparticles encapsulating diosgenin [31]. The combination of DSG and chemotherapeutic drug/Doxorubicin-based liposome was proven to be useful in the treatment of liver cancer with reduced toxicity and increased efficiency [32]. Nevertheless, many aspects of its potential are still undisclosed, which is why, in the present study, DSG was paired with resveratrol-rich PgnC *extract* in lipid nanodelivery systems, namely NLC, for which literature data are poor or limited. Consequently, and considering the pharmacological importance of the two phytochemical active principles, the current research monitors the synergism of PgnC extract with DSG for dual anti-inflammatory and antioxidant potential.

## 2. Materials and Methods

### 2.1. Materials

Polyoxyethylene sorbitan monolaureate (Tw20) and sorbitan monooleate (Sp80) as hydrogen peroxide and dimethyl sulfoxide (DMSO) were acquired from Merck (Darmstadt, Germany); glycerol monostearate (GMS) was obtained from Cognis GmbH (Monheim am Rhein, Germany); and cacao butter (CB) was purchased from a local drugstore. Regarding vegetable oils, linseed (Lo) and thistle oils (To), as well as the active herbals, Diosgenin/DSG 95% purity (3β,25R)-spirost-5-en-3-ol, C27H42O3) and *Polygonum cuspidatum*/PgnC (containing 50% resveratrol), were supplied by Organic Herb Inc. (Changsha, China). Tris[hydroxymethyl] aminomethane, 5-Amino-2,3-dihydro-1,4-phthalazinedione (Luminol), potassium persulfate, 2,2-azinobis-(3-ethylbenzothiazoline-6-sulfonic acid) (ABTS), and 6-hydroxy-2,5,7,8-tetramethyl chroman-2-carboxylic acid (Trolox) were purchased from Sigma Aldrich (Darmstadt, Germany).

The standardized cell line, EA.hy926 (vascular endothelial cells) bought from American Type CultureCollection (ATCC), was used for the evaluation of NLC cytotoxicity employing two assays: the MTS Tetrazolium assay (MTS) and real-time cell analysis (RTCA).

### 2.2. Preparation of PgnC and/or DSG-Loaded Nanostructured Lipid Carriers

PgnC/DSG-loaded nanostructured lipid carriers were fabricated via the melt-emulsification technique followed by high-pressure homogenization, the procedure of which is described in detail by Coc et al. [33]. In brief, the lipid phase (10% to the total mass) containing solid lipid (GMS and cacao butter, 7%) and liquid lipid (To and Lo, 3%), as the aqueous phase (containing 2.5% surfactants mixture) were heated up at 73oC, until complete melting and solubilization. Phytochemical active principles were added right after this step, based on the compound’s affinity, DSG (0.5%) into the lipid phase and PgnC extract (0.2%) into the aqueous phase. Then, these were mixed and kept at the same temperature for 15 min under constant stirring in order to obtain the pre-emulsion. Afterwards, the hot coarse emulsion was subjected to high-sheer homogenization (HSH) (High Shear Homogenizer PRO250 type, Conecticut, USA) at 13,000 rpm for 1 min, then to high-pressure homogenization (HPH) (APV 2000 Lab Homogenizer, Norderstedt, Germany) for 6 homogenization cycles, reaching 500 bar. Finally, the obtained nanoemulsions were cooled at room temperature and freeze-dried using a Christ Alpha 1-2 LD Freeze Dryer, Osterode am Harz Germany (−55 °C, 54 h), obtaining solid Polygonum cuspidatum-loaded nanostructured lipid carriers.

### 2.3. Determination of Particle Size and Polydispersity Index

The average particle size and polydispersity index (PdI) of synthesized nanoemulsions were assessed using a dynamic light scattering technique (DLS) (Zetasizer Nano ZS, Malvern Instruments, Malvern, UK) at room temperature (25 °C) with a fixed angle of 90°. The scattering intensity was adjusted by multiple dilutions (100-fold dilution) of the NLC dispersion prior to analysis. Data quantification was achieved using the Stokes–Einstein equation. Measurements were replicated three times.

### 2.4. Morphological Aspects of NLC

The morphological aspects of the optimum PgnC/DSG-loaded NLC samples were studied using scanning transmission electron microscopy (Hitachi HD 2700 Scanning Transmission Electron Microscope, Tokyo, Japan) in bright field mode. For STEM analysis, the dispersions were diluted in 20 mL distilled water and further positioned on appropriate Cu TEM grids provided with carbon-slight pellicle. The images were obtained using different detectors (SE—secondary electrons; ZC—phase contrast; and TE—transmission electrons) at different magnifications: ×50 K, ×80 K, and ×100 K.

### 2.5. Zeta Potential Analysis

The Zeta potential (ξ) or electrokinetic potential, measuring the surface charge of nanoparticles in dispersions, was investigated with the Zetasizer Nano ZS, Malvern Instruments, Malvern, UK. Before analysis, samples were diluted using ultra-purified water adjusted with 0.9% sodium chloride solution and freshly prepared in order to attain 50 µS/cm. Samples were measured in triplicate, and the results were expressed as the average ± S.D. Using Helmoltz–Smoluchowski Equation (1), these were rapidly evaluated:

(1) $$\xi =EM\times \frac{4\pi \eta }{\varepsilon }$$

*ξ* is the zeta potential, *EM* is the electrophoretic mobility, *η* is the viscosity of the dispersion medium, and *ε* is the dielectric constant.

### 2.6. Differential Scanning Calorimetry (DSC)

The thermal behavior of the freeze-dried NLC was evaluated using a differential scanning calorimeter Netzsch DSC 204F1 Phoenix, Selb, Germany. Using aluminum pans, 20 mg of sample (free or/and co-loaded NLC) was sealed in and then heated from 25° until 100 °C, maintaining a constant 10 °C/min cooling/heating rate, using nitrogen flow (40 mL/min). As a reference, an empty aluminum pan was used.

### 2.7. The Entrapment Efficiency of Phytochemicals

The entrapment efficiency of PgnC, namely resveratrol, was determined using a spectrophotometric UV method. A total of 0.5 g of lyophilized NLC, free and co-/loaded, was weighed and dispersed in 1 mL ethanol, being further subjected to 13,000 rpm for 15 min using a centrifuge (Sigma 2K15, Osterode am Harz, Germany). The resultant supernatant containing the un-encapsulated phytochemical active principle was analyzed using a UV–VIS–NIR Spectrophotometer Type V670, (Jasco, Tokyo, Japan). Resveratrol analysis was performed at λ = 307 nm. The calibration curve of resveratrol was obtained by measuring the absorbance of 7 known resveratrol concentrations in the range 0.5–5 μg/mL. The regression equation determined was y = 0.1521x + 0.004, with an R^2^ value of 0.999. Following the absorbance spectral analysis, the un-encapsulated percentage of resveratrol was calculated from the regression equation, taking into consideration the initial amount added into the NLC formulations.

Regarding the quantification of DSG entrapped in NLC, this was achieved using a chromatographic method. Samples were prepared similar to those used for PgnC EE % analysis, and they were then subjected to a Jasco 2000 liquid chromatograph equipped with a Nucleosil C18 column (25 × 0.4 mm) and a UV detector at λ = 203 nm. The mobile phase made of CH_3_CN: H_3_PO_4_ (0.5% *v*/*v*) was set at a 1 mL/min flow; retention time was 13.54 min for DSG. Also, for DSG, the concentration range of the calibration curve was 20–200 µg/mL, with R^2^ = 0.9989.

The concentration of un-encapsulated phytochemical active principle was determined using the initial concentration, as that quantified in the supernatant using the UV-VIS/HPLC method, in terms of entrapment efficiency:

(2) $$EE\;\left(\%\right)=\frac{M_{i}-M_{s}}{M_{i}}\times 100$$

*M_i_* = initial mass of the loaded phytochemical active principle; *Ms* = mass of phytochemical active principle found in the supernatant phase.

### 2.8. In Vitro Antioxidant Activity of NLC

#### 2.8.1. Chemiluminescence Assay

In order to quantify the scavenging capability of the newly produced nanosystems against short-life free radicals, a Chemiluminometer Turner Design TD 20/20, Sunnyvale, CA, USA, was employed. The chemiluminescent system was composed of luminol 10^−5^ M—an organic compound which emits light when oxidized—being converted into an excited amino-phthalate dianion and H_2_O_2_ (10^−5^ M) in a buffer solution Tris–HCl (pH = 8.6).

The antioxidant activities exhibited by the free and co-/loaded samples were measured using:

(3) $$\mathrm{AA}\;\left(\%\right)=\frac{I_{0}-I_{s}}{I_{0}}\times 100$$

*I*_0_ = the maximum reference intensity after 5 s; *Is* = the maximum sample intensity after 5 s.

#### 2.8.2. ABTS Assay

The scavenging capability against long-life radicals of nanosystems was evaluated using the ABTS spectrophotometric assay. The ABTS^.+^ radical was generated following the interaction between ABTS solution (7 mM) and K_2_S_2_O_8_ in water (2.45 mM), prepared 16 h before handling and maintained in the dark at room temperature. In brief, ABTS^.+^ cation, radical, changes colour after being reduced by an antioxidant, namely the re-suspended freeze-dried NLC. The measurement was performed at 734 nm. Samples’ preparation involved the addition of 3 mL ABTS^.+^ solution into 1 mL NLC solution (0.05 g lyophilized nanocarriers for DSG determination, respectively 0,1 g PgnC-loaded NLC in 10 mL ethanol for resveratrol quantification) ) and 2 mL of ethanol, mixed in 5 mL volumetric flasks. The blank solution was prepared identically by replacing the volume of the sample with the reference solution. The absorbance of the samples was measured using a UV–VIS–NIR Spectrophotometer Type V670, (Jasco, Tokyo, Japan), after exactly 4 min, using ethanol as a reference. The scavenging capability, expressed as inhibition (*Inh* %), was calculated according to:

(4) $$Inh\;\left(\%\right)=\frac{A_{0}-A_{s}}{A_{0}}\times 100$$

*A*_0_ = the absorbance of the blank (un-scavenged radical cation solution); *As* = the absorbance after the addition of the NLC solution (antioxidant solution).

### 2.9. In Vitro Controlled Release

The in vitro release experiments on phytochemical active principles captured in NLC were performed using the dialysis bag diffusion method. A total of 5 mL of aqueous dispersion of loaded nanocarrier was poured into a dialysis bag (MWCO 12–14 kDa) and immersed into 50 mL of ethanol:phosphate buffer solution, pH 6.4 = 70:30, representing the release medium. The receptor medium was chosen to provide the best results for both, diosgenin and PgnC. The process was conducted under controlled temperature, 37 °C, and continuous stirring at 300 rpm. A total of 500 µL of sample was collected each hour and replaced with fresh receptor medium, at 37 °C, over a period of 8 h. The amount of released phytochemical active principles was analysed using two methods: HPLC for diosgenin quantification and UV-Vis spectrometry for PgnC, specifically resveratrol (see Section 2.7).

### 2.10. In Vitro Assessment of Cell Viability and Cytotoxicity

#### 2.10.1. MTS Cell Viability Assay

The viability, in vitro, of each cell was studied through MTS colorimetric assay (MTS, a tetrazolium salt, can be converted into formazans in the presence of active cells). In brief, using a CellTiter 96 Aqueous One Solution Cell Proliferation assay (Promega, Madison, Wi, USA), 1 × 10^4^ cells/well were seeded into 96-well microtiter plates with a flat bottom (Falcon, Suzhou, China) and were cultured for 24 h in 100 μL of medium. Following the supernatant removal, EA.hy926 cells were, for a time, treated with different concentrations of loaded and co-loaded DSG and PgnC for 24 or 48 h, namely 3.125, 6.25, 12.5, 25, 50, 100, and 200 µg/mL. After the specific time, 20 μL of reagent composed of MTS [3-(4,5-dimethylthiazol-2-yl)-5-(3-carboxymethoxyphenyl)-2-(4-sulfophenyl)-2H-tetrazolium inner salt] and PES (phenazine ethosulfate) were added into each well with 100 µL cells (final concentration of MTS of 0.33 μg/mL) and incubated for 1–4 h at 37 °C, shaking it every 15 min. The absorbance of the newly formed formazans was measured at a wavelength of λ = 492 nm using a Dynex plate reader (DYNEX Technologies MRS, Chantilly, VA, USA). All MTS cell viability experiments consisted of three independent replicates. Cell viability was determined using the equation

(5) $$\;\mathrm{Cell}\;\mathrm{viability}\;\left(\%\right)=\frac{{\mathrm{Abs}}_{\mathrm{treated}\;\mathrm{cells}}-{\;\mathrm{Abs}}_{\mathrm{culture}\;\mathrm{medium}}}{{\mathrm{Abs}}_{\mathrm{untreated}\;\mathrm{cells}}-{\;\mathrm{Abs}}_{\mathrm{culture}\;\mathrm{medium}}}\times 100\;$$

Abs = the absorbance of treated/untreated and culture medium.

#### 2.10.2. Cytotoxicity vs. Cell Viability by Real-Time Cell Analysis (RTCA)

Real-time cell analysis relies on xCELLigence technology and an RTCA-DP analyser (ACEA Biosciences, San Diego, CA, USA) for the determination of cell viability vs. cytotoxicity. In short, 1 × 10^4^ EA.hy926 normal cells were seeded in 100 μL culture medium in 16-cell E-Plates (ACEA Biosciences), then transferred in the xCELLigence DP-System, before being placed in a 5% CO_2_, humidified incubator. The growth cell curves were recorded on the xCELLigence System in real time via the electronic sensors measuring cell impedance, which will furthermore be referred to as the cell index (CI). When CI value exceeds 1.0, increasing concentrations of PgnC/DSG-loaded NLC are placed over and live cells were reported [34].

### 2.11. The Anti-Inflammatory Activity Using ELISA Assay

ELISA kits (“Ready-Set-Go”Affimetrix eBioscience, Thermo Fisher Scientific, Waltham, MA, USA) were used for the determination of the level of proinflammatory cytokines in order to express the anti-inflammatory activity of NLC systems. In view of capturing the specific antibodies, a coating step was conducted consisting of an overnight incubation at 4 °C in the 96-well plates. Further, following the washing with PBS-Tween 20 (0.05%) solution, the standard and loaded nanocarriers were added and incubated overnight at 4 °C. the next day, after washing again with PBS-Tween 20 (0.05%), incubation for the detection of specific antibodies was performed at room temperature (RT) for 1 h. The Avidin-HRP enzyme was added (30 min, RT), then the TMB substrate solution (15 min, RT) in order to identify the target molecules. Before the previously mentioned steps, 2–3× washes were performed. The addition of (NH_3_)_2_SO_4_ solution concluded the process. The optical densities were measured spectrophotometrically at 450 nm. Eight concentration points attained by 1:2 serial dilutions, starting from 500 pg/mL for IL-6 and 1000 pg/mL for TNF-a, have served for the standard curves’ design. The analysis of the level of IL-6 and TNF-α cytokines was evaluated in EA.hy926 cell culture supernatants, with or without induced-inflammation, at the end of treatments with NLC and/or H_2_O_2_. Conditioned media was collected and centrifuged (30 min, 400× *g*), and the supernatants were stored at −80 °C until the ELISA assay was performed. Values were drawn from the standard curves plotted for each cytokine, being expressed in pg/mL.

### 2.12. Statistical Analysis

The data, expressed as their mean value ± standard deviation (SD), are the average of three independent experiments. Statistical data analyses were performed using GraphPad Prism 7 (GraphPad Software Inc., La Jolla, CA, USA). Unpaired, two-tailed *t*-test and one-way ANOVA were employed to compare the treated with the control group. Data with *p* < 0.05 were considered significant statistically, whereas *p* > 0.05, NS = not significant.

## 3. Results and Discussion

### 3.1. Size, Stability, and Chemical Characteristics of Individual and Dual- NLC-To/Lo–Phytochemical Actives

**Figure 1:**
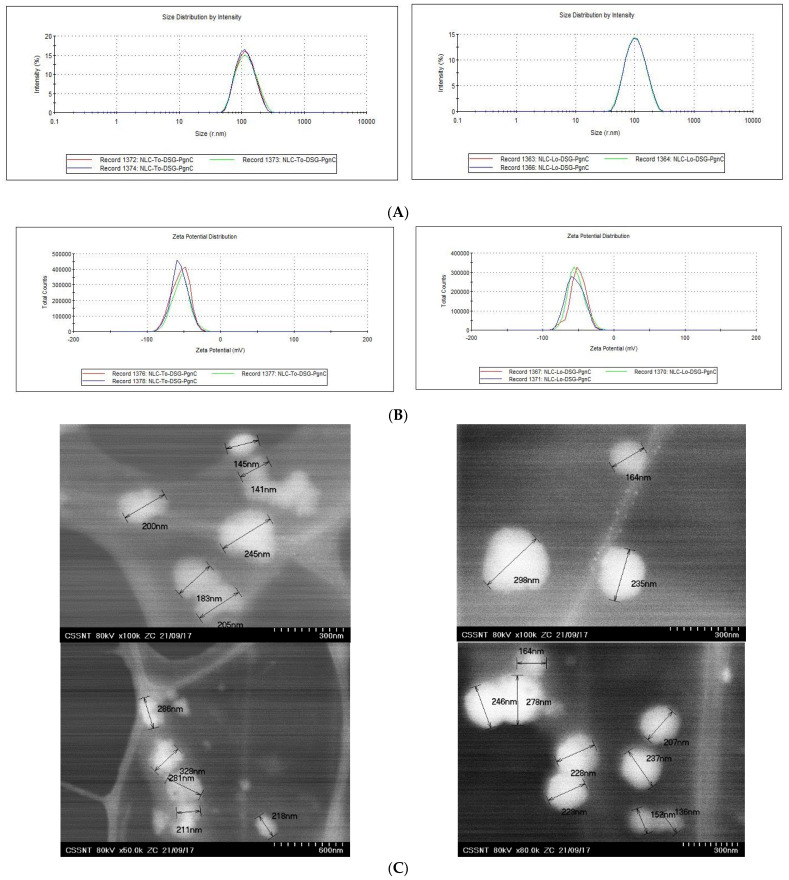
Size distribution (**A**); zeta potential (**B**); and morphology of the particles (**C**) for the NLC containing dual herbal principles (DSG and PgnC).

The integrity and stability of the lipid nanocarriers can be controlled by selecting the appropriate type of lipids, the nature and concentration of the emulsifier(s) used to stabilize the initial oil droplets, and the cooling conditions. In order to obtain lipid nanocarriers with appropriate size and physical stability characteristics, a lipid mixture consisting of glyceryl monostearate, cocoa butter, and thistle oil/linseed oil was used in a weight ratio of 1.16:1.16:1) paired with a surfactant mixture consisting of Tween 20 and Span 80 (weight ratio 2.125:1). The encapsulation of a single herbal principle (*PgnC* or DSG) led to average diameter values lower than 210 nm, slight decreases being reported in the case of NLC prepared with linseed oil (Table 1). The decreasing in the average diameters/Zave of NLC prepared with To or Lo may be the result of the difference in viscosity of the two vegetable oils, a difference which impacts the processing through homogenization/HPH (ex: linseed oil has an absolute viscosity of 33.1 cP, while thistle oil has a viscosity of 51.2 cP at 25 °C). The variable content of unsaturated fatty acids/FAs, ω-3, -6 and 9 could influence the internal arrangement of hydrophobic tails of FAs from To, Lo, MSG, and CB. For example, linseed oil is primarily composed of fatty acids such as α-linolenic acid C18:3, w3 (53%); oleic acid C18:1, ω-9 (20.8%); and linoleic acid, C18:2, ω-6 (13.1%). Among the fatty acids present in thistle oil, linoleic acid, C18:2, ω-6 is the most abundant (52–54%), followed by oleic acid, C18:1, ω-9 (27–29%) and palmitic acid (7–8%). The lipid nanocarriers carrying dual herbal principles (NLC-DSG-PgnC) had average diameters of 218.2 nm ± 0.89 and 193.9 nm ± 2.95, respectively (Table 1 and Figure 1A). According to Figure 1B and the PdI values (Table 1), almost all of the lipid nanocarriers had a relatively narrow size distribution; the polydispersity index values ranged between 0.153 and 0.219 (for NLC-To systems) and 0.144 ± 0.183 (for NLC-Lo systems). The distribution of the dual NLC within a narrow size range with polydispersity index values of 0.114 ± 0.02 for NLC-To-DSG-PgnC and of 0.152 ± 0.02 for NLC-Lo-DSG-PgnC (Figure 1A). According to the TEM analysis, the nanocarriers have a specific spherical morphology, with spheres of diameters between 100 and 250 nm (Figure 1C). All of the lipid nanocarriers loaded with herbal active principles showed values of electrokinetic potential greater than −46.5 mV (Table 1 and Figure 1B), which suggests the existence of a superficial charge distribution suitable for ensuring high stability of the lipid nanoparticles over time. The occurrence of the electronegative charges can be explained based on the distribution of free fatty acids (from a lipid blend) at the oil–water interface with the exposure of the carboxyl groups to the outside of the NLC.

***Entrapment efficiency of newly loaded and co-loaded NLC-To/Lo-herbal actives***

The results of the quantitative determinations performed by HPLC and UV-Vis highlighted the higher affinity of Diosgenin for the lipid mixture formed by the two solid lipids with linseed oil (e.g.,: EE_NLC-Lo-DSG_ = 91.6% ± 0.3 vs. EE_NLC-To-DSG_ = 86.6% ± 2.50). One potential explanation could be attributed to the better solubility of lipophilic DSG in linseed oil, which presents a lipid profile rich in unsaturated fatty acids, the most abundant being linoleic, linolenic, and oleic acids. Referring to PgnC entrapment, an influence of the oils used could also be seen in the encapsulation efficiency percentages (e.g.,: 64.10 ± 1.50 vs. 73.00 ± 1.30). The composition of PgnC extract, rich in hydrophilic phytoconstituents, helped in obtaining moderate encapsulation percentages for dual NLCs, the encapsulation efficiency values being 64.1 and 57% for PgnC and 87% and 81% for the lipophilic/DSG active (Table 1).

***Structural changes in NLC-To/Lo following the incorporation of herbal actives***

**Figure 2:**
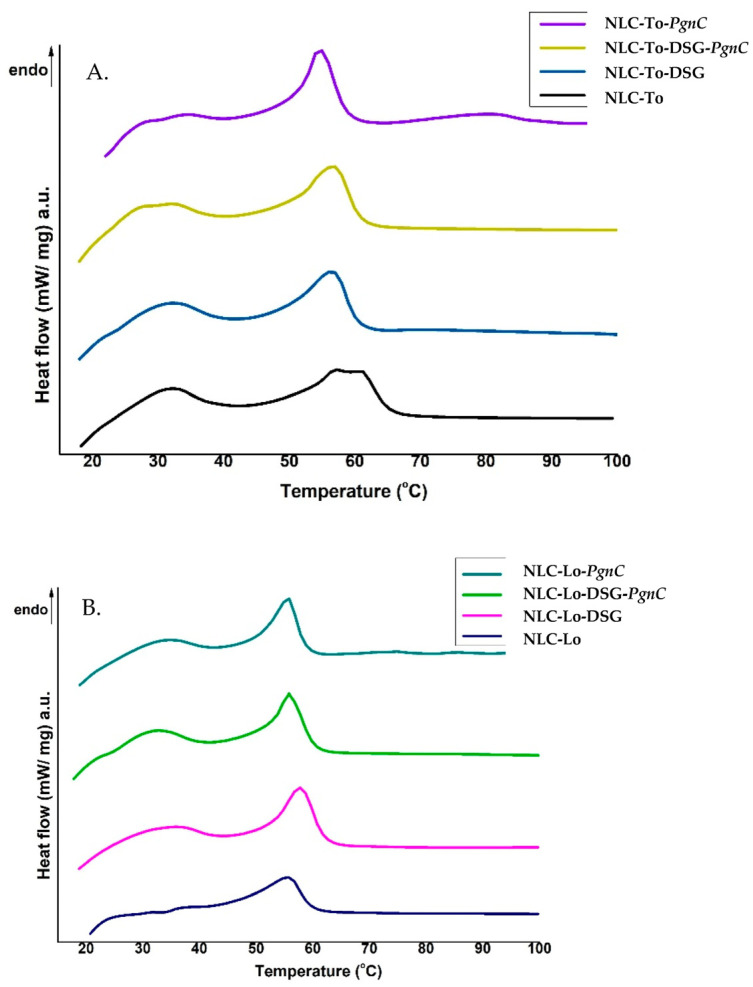
DSC thermograms of NLC prepared with thistle oil (**A**) and linseed oil (**B**), loaded with DSG, PgnC, and or a mixture of DSG and PgnC.

Differential scanning calorimetry (DSC) was used to follow the structural changes to lipid nanocarriers before and after the capture of herbal active principles. Structural alterations to lipid nanocarriers are accompanied by heat exchanges, e.g., the uptake of heat during melting or the emission of it during crystallization. These heat exchanges during controlled temperature programs allow for conclusions to be drawn as to the structural properties of the NLC. The signals obtained in DSC measurements, by means of the heat flow as a function of time and temperature, for NLC loaded with herbal actives were compared with those of unloaded-NLC (Figure 2). The melting process of the lipids, part of the two lipid cores of the NLC (prepared with Lo and To) occurred during one endothermic field with a temperature range of 60–67 °C. It is known that GMS has a melting range varying from 57 to 65 °C, while cocoa butter has an endothermic peak around 36 °C [35]. The last one could be recognised as a weak shoulder around 33 °C.

The NLC loaded with DSG and herbal extract/PgnC led to a decrease in the melting point (signaled in DSC by peaks located between 55 and 60 °C). The bulk lipids (GMO, cocoa butter, and Lo or To oils) showed melting temperatures of 63.8 °C and 62.7 °C, respectively, while the NLC loaded with dual herbal actives registered a melting point of 57 °C and 56 °C, in NLC-DSG-PgnC prepared with To and Lo, respectively. A shift of the melting point towards lower temperatures with the decrease of the lipid particles’ diameters is known in the literature [36] and was also reported by Gokce et al. when they prepared SLN-resveratrol [37]. Lipids brought to the nanometric scale have a melting point below the melting temperature of bulk lipids (e.g., a decrease in m.p. by approx. 1–5 °C), due to the higher ratio between the specific surface area and the volume of particles with a smaller size [38]. The decrease in melting enthalpy (ΔHm, Table 2) identified in the case of NLC-herbal actives indicates a distortion of the internal lipid network similar to the distortion of the crystalline structure of bulk lipids, but in the case of NLC, it is more pronounced. The lower values of ΔHm indicate a massive disruption of the lipid core, in particular the less-ordered lipid core, favourable for a higher loading with active principle. As can be seen in Table 2, the lowest ΔHm values were recorded for the NLC that incorporates dual actives. In addition, the ΔHm value of unloaded NLC-Lo/To does not differ much from that of NLC-Lo-PgnC, while the values are significantly lower in the case of NLC-DSG-PgnC, e.g., ΔHm = 38.56 J/g for NLC-To, 36.66 J/g for NLC-Lo-PgnC, and 30.5 J/g for NLC-Lo-DSG-PgnC. These last aspects suggest (i) a lack of lipid core perturbation after PgnC loading and confirmation of the distribution of Polygonum cuspidatum into the non-ionic emulsifiers’ coating; (ii) NLC-herbal actives become sharper compared to unloaded-NLC, which reveals that there was an internal rearrangement of the lipid network after DSG encapsulation and confirms the molecular dispersion of the herbal active/DSG inside the lipid network.

### 3.2. In Vitro Cytotoxicity of NLC (MTS and RTCA Assays)

**Figure 3:**
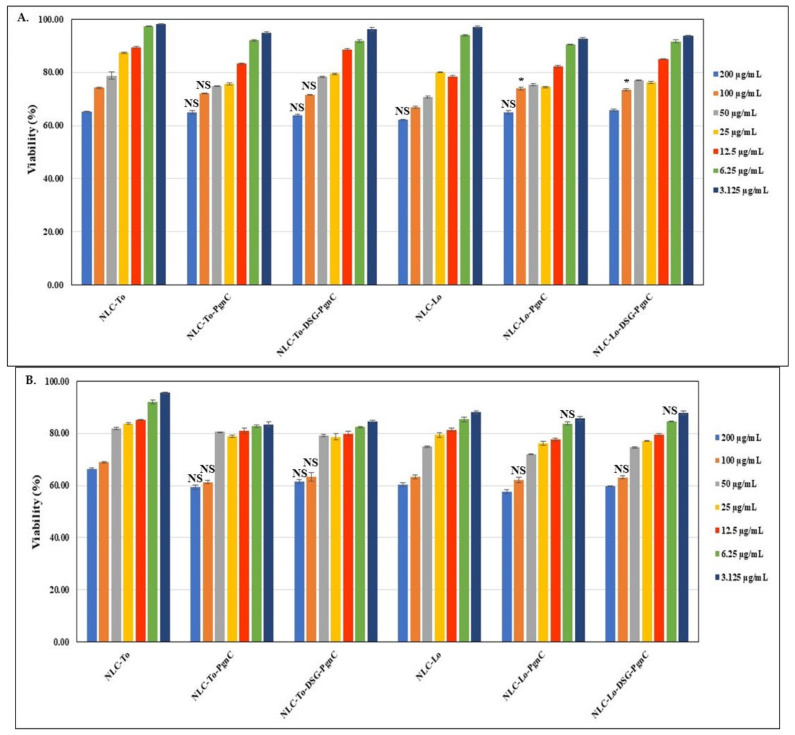
The effect of individual and dual nanocarriers, NLC-PgnC and NLC-DSG-PgnC, on the viability of normal EA.hy926 cells after 24 h (**A**) and 48 h (**B**) * *p* < 0.05, NS = not significant.

The toxicological profiles of the different individual-NLC (encapsulating only PgnC) and dual-NLC (carrying the two active principles—DSG together with PgnC) are presented in Figure 3. The MTS results revealed that, using NLC-phytochemical concentrations between 3.125 and 50 µg/mL, cell viability was maintained to values distributed between 70.6 and 98.3% (24 h treatment) and 72.1 and 96.7% (48 h treatment), which, according to ISO 10993-5:2009, indicates a lack of cytotoxicity induced by the treatment with NLC-To/Lo-PgnC and NLC-To/Lo-DSG-PgnC. The lipid nanocarriers prepared with thistle oil and linseed oil showed relatively similar cell viability on normal EA.hy926 cells, regardless of the type of encapsulated active principle. A slight tendency to decrease viability was observed for NLC-DSG, but cell viability in the case of NLC-dual remained relatively constant. For instance, a 48 h treatment with 50 µg/mL of NLC-To-DSG-PgnC ensured a cell viability of 79.2% ± 0.47 compared to the viability of 81.9 ± 0.47% determined for unloaded-NLC-To. Treatment for 24 h with concentrations of 50 µg/mL of NLC-Lo-dual led to an increase in cell viability, while the prolonged treatment of 48 h led to similar percentages of cell viability, e.g., 77 ± 0.18% NLC-Lo-DSG-PgnC vs. 70.6% ± 0.43 for unloaded-NLC-Lo (24 h treatment).

**Figure 4:**
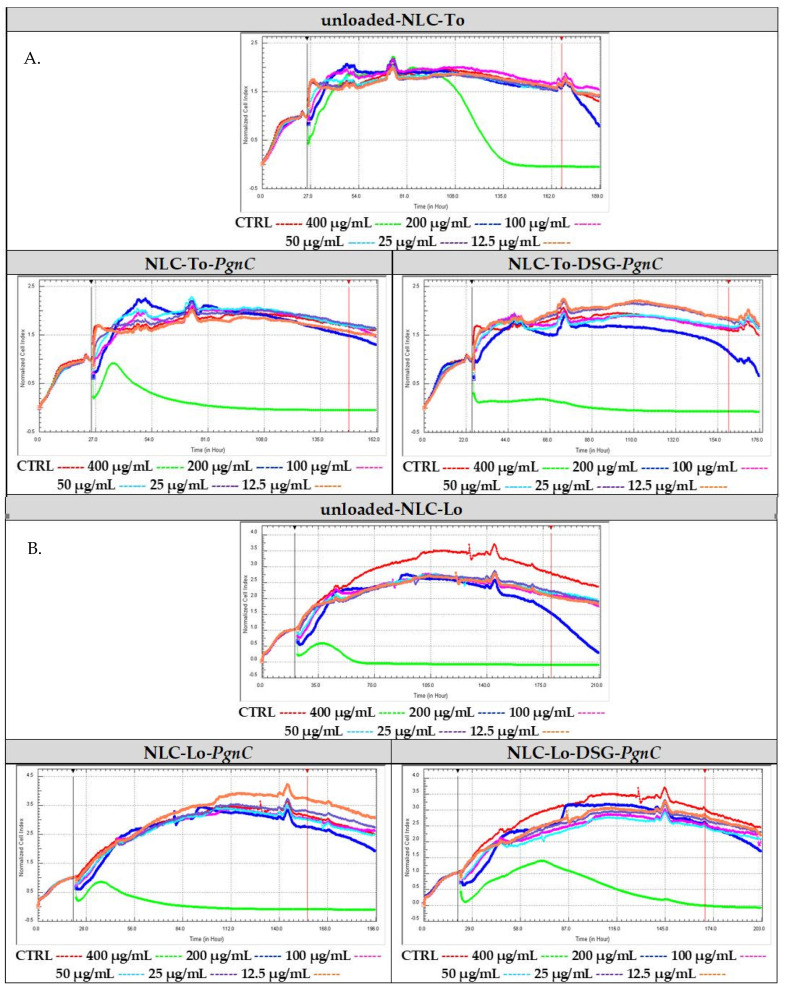
Cytotoxic vs. proliferative action induced by nanocarriers NLC-To-PgnC/DSG-PgnC (**A**) and NLC-Lo- PgnC /DSG-PgnC on normal cells EA.hy926 (**B**).

In order to compare in real time the proliferative capacity vs. cytotoxicity of the NLC-PgnC and NLC-DSG-PgnC on EA.hy926 cells, an RTCA assay was performed. For a more faithful highlighting of these parameters, the results were normalized compared to the untreated cells, as is represented in Figure 4 through the red curve. After evaluating the cytotoxicity vs. proliferation of normal EA.hy926 cells treated with different concentrations of NLC, it was observed that, at high concentrations (400 µg/mL and, in some cases, 200 µg/mL), viability decreases significantly, indicating an increase in cytotoxicity. Conversely, concentrations lower than 100 µg/mL ensure cell viability percentages comparable to those of untreated cells (control). This last aspect demonstrates the safety of NLC concentrations between 25 and 100 µg/mL.

Regarding the lipid nanocarriers intended for the distribution of Diosgenin and PgnC, cell proliferative indices were observed for concentrations of 12.5 and 25 µg/mL (for NLC-To-DSG-PgnC, Figure 4A). Concentrations of 50 µg/mL ensured a biocompatible effect similar to that of untreated cells in the case of NLC-To-PgnC and NLC-To-DSG-PgnC (Figure 4A). Referring to NLC prepared with linseed oil, a decrease in the biocompatibility of cells subjected to in vitro analysis was observed (Figure 4B). Only for NLC-Lo-PgnC was a high biocompatibility induced—valid for the 12.5 µg/mL—while for NLC-Lo-DSG-PgnC, the distribution of the cellular index was below the control level (untreated cells, Figure 4B).

### 3.3. In Vitro Determination of Antioxidant Activity

**Figure 5:**
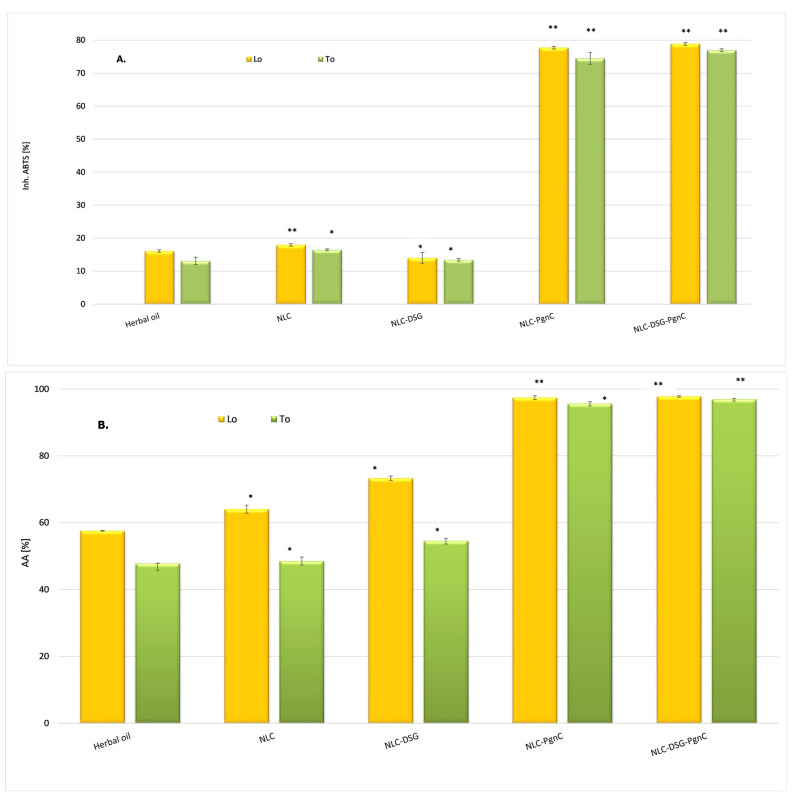
(**A**). ABTS + cationic radicals capture the activity of unloaded-NLC and NLC-DSG-PgnC (by TEAC method). (**B**). The ability to annihilate ROS species by unloaded-NLC and NLC-DSG-PgnC (by chemiluminescence assay). * *p* < 0.05; ** *p* < 0.005; Data are expressed as mean ± SD, *n* = 3, herbal oil vs. other groups.

Oxidative stress is known to contribute to carcinogenesis, with free radicals causing lipid peroxidation and DNA damage. ROS can interact with the polyunsaturated phospholipids on cell membranes, causing subsequent generation of products that could detrimentally react with cellular proteins and DNA [39]. The correct form is: The ability to inhibit long-live ABTS•+ radicals (TEAC method) indicated that NLC-DSG exhibited a modest radical scavenging activity, between 13 and 16%. On the other hand, NLC that encapsulated PgnC via the individual or dual approach presented a clearly superior ability to capture cationic radicals, this being between 74.5 and 77.9% (Figure 5A). This behaviour can be explained based on the composition of PgnC extract, which contains phytochemicals belonging to flavonoids, quinones, stilbenes, coumarins, and lignans [18], compounds with known antioxidant properties. Pan et al. evaluated the DPPH radical scavenging activity over time and observed a superior antioxidant effect of the ethanolic extract of PgnC superior relative to resveratrol, activity correlated with the high content of polyphenols and flavones present in the composition of this extract [40].

By testing the ability to inhibit the oxygenated free radicals (ROS) generated in the chemiluminescent system (through reaction between luminol and hydrogen peroxide), it was observed that the antioxidant activity was amplified in the case of NLC categories loaded with PgnC, e.g., 95.65 ± 0.7% and 97.44 ± 0.58% for NLC-To-PgnC and NLC-Lo-PgnC, respectively, (Figure 5B). By the simultaneous co-optation of the two herbal principles—PgnC extract and DSG—in the lipid nanocarriers, no significant increase in the ability of NLC to capture ROS radicals was observed, the antioxidant activity values being maintained at an equally high level, e.g., 97.0 ± 0.58% and 97.78 ± 0.23% for NLC-DSG-PgnC prepared with To and Lo, respectively. These results underline the non-involvement of DSG in stopping oxygenated free radicals [41], while the promotion of effective antioxidant activity can be attributed to the two vegetable oils and, implicitly, to the advantages of omega-6 and -9 fatty acids from To and Lo in capturing free radicals.

### 3.4. In Vitro Determination of Phytochemicals Co-Delivery

**Figure 6:**
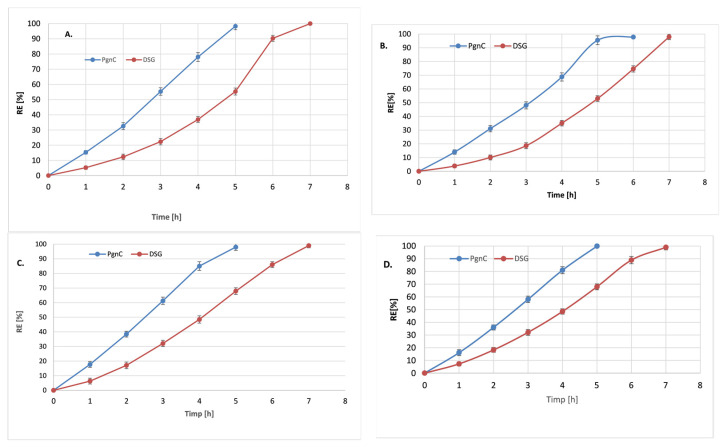
Release profiles of resveratrol and DSG from NLC-*To*-phytochemical active (**A**), NLC-*To*-dual phytochemical actives (**B**), NLC-*Lo*-phytochemical active (**C**), and NLC-*Lo*-dual phytochemical actives (**D**).

The release feature of the individual NLC revealed a rapid release of the main compound from PgnC. Starting from the first hour of the release study, percentages of 15.3% and 17.7% resveratrol were determined for NLC-To/Lo-PgnC, a total release of the polyphenol being achieved after 5 h (Figure 6A). A potential explanation of this behaviour could be associated with the preferential distribution of PgnC in the surfactant coating with which it forms weak bonds (hydrogen bonds). However, for Diosgenin present in nanocarrier systems, different release behaviour was observed. Although the receptor environment favoured release in a relatively fast approach for both phytochemicals, nevertheless, in the case of DSG, it was observed that after 5 h, the degree of release reached a value of 55% and 67% DSG from NLC-To/Lo-DSG compared to 100% PgnC from NLC-To/Lo-PgnC. The slower release of DSG can be explained by (i) the lipophilic character of DSG (the low solubility of DSG in the receiving environment can also be considered a determining factor); (ii) DSG entrapment into the nanocompartments of the lipid core (the exit of DSG from these nanocompartments and the passage through the complex lipid networks determined that DSG exhibits a slower release compared to resveratrol) [42].

The dual NLC-To delivery systems assured a much slower release of both phytochemicals, a phenomenon that was more pronounced in the case of the PgnC extract compared to the NLC loaded with only one of the two active phytochemicals. For example, individual nanocarrier systems prepared with linseed oil released ~87% PgnC after 4 h of experiments (Figure 6C), while 80% PgnC was released from dual-NLC (Figure 6D). In the case of DSG release from the dual lipid distribution systems, after 4 h, relatively equal amounts of DSG were determined in the receiving environment: 48% from the dual system vs. 47% DSG from the individual-NLC. This uniform release can be attributed to the good adaptability and affinity of DSG in the lipid core formed by cocoa butter, glyceryl monostearate, and vegetable oils—To or Lo. A total release of DSG from NLC systems occurs after 7 h, regardless of the category of NLC (individual or dual).

### 3.5. Kinetic Modelling Studies

The kinetic parameters (rate constant k, correlation coefficient R^2^, and release coefficient n) obtained by fitting the data obtained from the in vitro release of DSG and PgnC extract (containing 50% resveratrol) with the five kinetic models are presented in Table 3 and Figure 6. Analysing the kinetic parameters for each mathematical model, it is observed that the best correlation coefficient, R^2^, was obtained in the case of the Peppas–Korsmeyer model, both for DSG and PgnC in NLC-individual or NLC-dual systems. The release coefficient, n, contained in the interval 0.301 < *n* < 0.459 indicates the existence of a Fickian diffusion type mechanism with a spherical geometry of the lipid delivery system [43]. In both situations, the spherical morphology of the lipid nanocarriers was confirmed by TEM microscopy. The release behaviour of the two phytocompounds is also supported by the values of the release rate constants (Table 3). For instance, for the release of Pgn C from NLC-To, they are 1.8 times higher in the case of NLC-individual and 2.13 times in the case of NLC-dual systems. Notable is the fact that there were no significant differences between the values of the rate constants for the release of PgnC in the individual and dual NLC-Lo (2.891 h^−1^ vs. 2.786 h^−1^).

The differences observed in the release of Diosgenin and resveratrol (from PgnC extract) can be explained based on the differing solubility of the phytocompounds and their ability to form weak interactions with the coating surfactants. Thus, a fast release of PgnC may be due to some more-hydrophilic phytoconstituents present in the PgnC *extract*, which confer an advanced solubility in the receptor medium. Also, the lipophilic character of DSG allows it to be more efficiently incorporated into the lipid matrix, filling a significant percentage of the total loading capacity of the lipid core, while PgnC is mainly hosted by the surfactant coating.

### 3.6. In Vitro Quantification of Anti-Inflammatory Action

**Figure 7:**
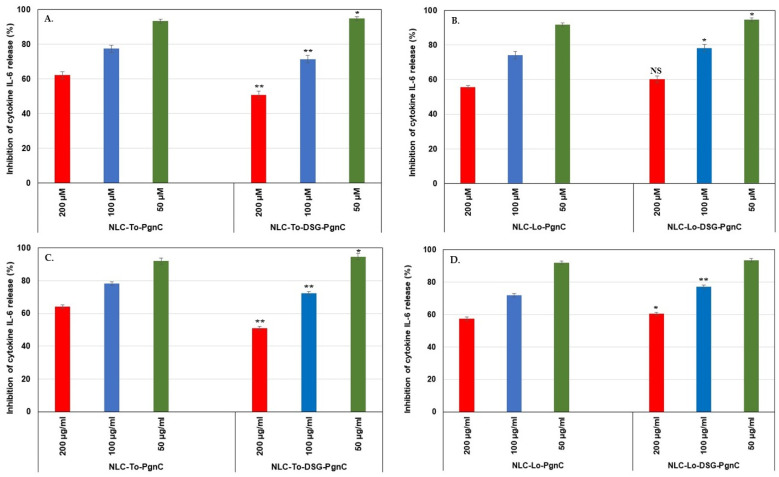
Evaluation of treatments with NLC-PgnC and NLC-DSG-PgnC on the release of IL-6 cytokine by normal EA.hy926 cells after 24 h (**A**,**B**) and 48 h (**C**,**D**) of treatment, using ELISA assay. * *p* < 0.05; ** *p* < 0.005; NS = non-significant. Data are expressed as mean ± SD, *n* = 3; NLC-To/Lo-PgnC vs. other groups.

**Figure 8:**
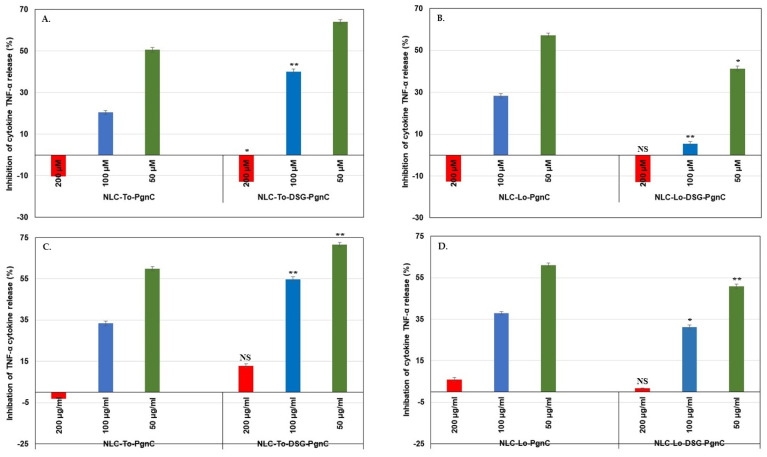
Evaluation of treatments with NLC-PgnC and NLC-DSG-PgnC for the release of TNF-α cytokine by normal EA.hy926 cells after 24 h (**A**,**B**) and 48 h of treatment (**C**,**D**) using an ELISA assay. * *p* < 0.05; ** *p* < 0.005, NS = non-significant. Data are expressed as mean ± SD, *n* = 3 NLC-To/Lo-PgnC vs. other groups.

Cytokines are glycoproteins capable of modulating the inflammatory response. Interleukin 6 (IL-6) and tumor necrosis factor alpha (TNF-α) are considered the critical pro-inflammatory cytokines involved in the pathogenesis of inflammatory diseases [44,45]. As such, the inhibition of these cytokines and related signaling pathways is considered a target for the development of therapeutics for various diseases. When studying the in vitro behavior of NLC-PgnC and NLC-DSG-PgnC in terms of the expression of the pro-inflammatory cytokines IL-6 and TNF-α, a strong anti-inflammatory effect was highlighted. According to the ELISA results, the treating of EA.hy926 cells with different concentrations of NLC-To/Lo-phytochemical actives resulted in a decreased production of pro-inflammatory cytokines TNF-α and IL-6 (Figure 7 and Figure 8). For instance, concentrations of 50 µg/mL NLC-PgnC and NLC-DSG-PgnC ensured a promising capacity of inhibition towards pro-inflammatory cytokine IL-6, e.g., 93.4 and 94.9% (after 24 h, Figure 7A) and, respectively, 92 and 94.7% (after 48 h, Figure 7C) for NLC-To; 91.7 and 94.6% (after 24 h, Figure 7B), and, respectively, 91.9 and 93.3% (after 48 h, Figure 7D) for NLC-Lo.

The superiority of the developed NLC-phytochemicals described in this work lead to the achievement of a doubling of the inhibition percentages in case of the pro-inflammatory cytokine TNF-α (Figure 8). For instance, the inhibition of TNF-α release was more pronounced for dual-NLC compared to single-NLC. NLC-*PgnC* assured an inhibition of 64% (at 24 h, Figure 8A), while for NLC-DSG-*PgnC*, 71.6% inhibition (after 48 h, Figure 8C) has been recorded. NLC-DSG has led to low inhibition values, i.e., 22.7% and 37.8% inhibition, after 24 h and 48h of treatment. A low decrease in the ability to inhibit the release of TNF-α by NLC-Lo-DSG-PgnC was recorded during the same treatament periods (Figure 8B,D). Instead, the nanocarrier systems synthesized with Lo containing only PgnC maintained high values of TNF-α inhibition (e.g., 50.7% and 61.1% for 24 h and 48 h of treatment) [29].

## 4. Conclusions

Resveratrol-rich PgnC extract was paired into lipid nanodelivery systems with an herbal steroidal sapogenin, Diosgenin (DSG), in the present study as a means of simultaneously combating dangerous free radicals and inhibiting pro-inflammatory cytokines. PgnC extract-based lipid nanocarriers had a specific spherical morphology, with diameters between 100 and 250 nm, a narrow particle size distribution (with a polydispersity index of ~0.15), good stability (electrokinetic potential values greater than −46.5 mV) and appropriate entrapment efficiency (i.e., 65% for PgnC and 87% for DSG). Scanning calorimetry analysis reflected a massive disruption of the lipid core after the encapsulation of phytochemicals accompanied by a decreasing in melting enthalpy. The study demonstrated that NLC-DSG-PgnC did not affect the cytotoxicity on a vascular endothelial EA.hy926 cell line and synergistically induced the quenching of free oxygen radicals and inhibition of the long-live ABTS•+ radicals, thereby achieving better antioxidant in vitro effects than NLC alone.. According to MTS and RTCA assays, 50 µg/mL NLC-To-PgnC and NLC-To-DSG-PgnC ensure a biocompatible effect similar to that of untreated cells. The chemiluminescence results pointed out the non-involvement of DSG in stopping oxygenated free radicals; the antioxidant activity was maintained at an equally high level, higher than 97% for NLC-PgnC and for dual-NLC. On the other hand, the TEAC assay showed that NLC loaded with PgnC in an individual or dual approach presented a clearly superior ability to capture cationic radicals, this being between 74.5 and 77.9%. The dual-NLC assured a much slower in vitro release of both phytochemicals, more pronounced in the case of the PgnC extract, compared to the NLC loaded with only one of the two active phytochemicals. For instance, individual-NLC prepared with linseed oil released ~78% PgnC after 4 h of experiments while 67% PgnC was released from dual-NLC. Analysing kinetic parameters, the best correlation coefficient was confirmed in the Peppas–Korsmeyer model, both for DSG and PgnC in NLC-individual or NLC-dual systems.

The developed lipid nanocarriers carrying dual herbal principles demonstrated a promising inhibition capacity of pro-inflammatory cytokine IL-6, with a degree of inhibition between 91.9 and 94.9%. The major advantage of the NLC-DSG-PgnC was to achieve a doubling of the inhibition percentages in the case of the pro-inflammatory cytokine TNF-α. In this context, the inhibition of TNF-α release was more pronounced for dual-NLC compared to single NLC-PgnC, i.e., 64% inhibition (at 24 h) and 71.6% inhibition (after 48 h) in the case of NLC-To-DSG-PgnC compared to NLC-To-DSG (22.7% inhibition after 24 h and 37.8% inhibition after 48 h of treatment).

In conclusion, the safety profile of PgnC extract and Diosgenin along with the ability of NLC to simultaneously provide antioxidant and anti-inflammatory action add value to future experiments on this topic. The prospects of these lipid nanocarriers carrying dual herbal principles need to be carefully analyzed in the future in conjunction with in vivo animal studies to optimize their efficacy and safety. These natural products for various inflammatory pathologies may offer safer alternatives to current drugs.

## Figures and Tables

**Table 1:** Size, potential, and entrapment efficiency values determined for NLC-*PgnC*/DSG and NLC-DSG-*PgnC*.

Formulation	Z_ave_ (nm)	PdI	ξ (mV)	EE_DSG and PgnC_ *(*%)
NLC-TO	170.1 ± 2.97	0.153 ± 0.03	−46.5 ± 0.40	-
NLC-TO-DSG	207.1 ± 3.40	0.169 ± 0.00	−47.8 ± 0.85	86.59 ± 2.50
NLC-TO-*PgnC*	184.2 ± 2.72	0.219 ± 0.00	−64.2 ± 0.72	73.00 ± 1.30
NLC-TO-DSG-*PgnC*	218.2 ± 0.89	0.114 ± 0.02	−54.8 ± 1.00	63.30 ± 3.71
				80.98 ± 0.15
NLC-LO	150.5 ± 2.19	0.144 ± 0.00	−48.0 ± 1.60	-
NLC-LO-DSG	200.5 ± 1.72	0.171 ± 0.01	−47.9 ± 0.96	91.64 ± 0.31
NLC-LO-*PgnC*	164.3 ± 1.87	0.183 ± 0.01	−55.4 ± 2.21	64.10 ± 1.50
NLC-LO-DSG-*PgnC*	193.9 ± 2.95	0.152 ± 0.02	−52.8 ± 2.15	56.60 ± 1.21
				86.98 ± 1.10

**Table 2:** Corresponding parameters of DSC obtained for NLC loaded with herbal actives.

Analysed Sample		T_m_ (°C)	ΔH_m_ (J/g)
1	Physical blend (GMS + CB + Lo)	63.8	46.1
2	NLC-Lo	55.5	38.56
3	NLC-Lo-DSG	59.8	33.98
4	NLC-Lo-PgnC	55.4	36.66
5	NLC-Lo-DSG-PgnC	56.0	30.5
6	Physical blend (GMS + CB + To)	62.7	47.15
7	NLC-To	60.0	39.47
8	NLC-To-DSG	58.5	32.01
9	NLC-To-*PgnC*	54.6	38.51
10	NLC-To-DSG-*PgnC*	57.0	25.08

**Table 3:** Kinetic parameters obtained during in vitro DSG and Pgn release from individual and dual NLC-To/Lo-herbal actives.

Formulations	Phyt.	Order 0		Order 1		Higuchi		Hixson-Crowell		Peppas-Korsmeyer		
		$\%R=k_{0}t$		$\ln(100-\%R)=k_{1}t$		$\%R=k_{2}\surd t$		$\sqrt[{3}]{100-\%R}=k_{3}t$		$\%R=k_{4}\sqrt[{n}]{t}$		
		R^2^	k_0_	R^2^	k_1_	R^2^	k_2_	R^2^	k_3_	R^2^	k_4_	*n*
NLC-To-DSG	DSG	0.904	14.133	0.686	0.336	0.852	54.314	0.882	0.019	0.984	1.524	0.332
NLC-To-PgnC	PgnC	0.994	20.710	0.755	0.710	0.978	68.046	0.640	0.138	0.999	2.711	0.427
NLC-To-DSG-PgnC	DSG	0.937	14.104 *	0.830	0.210	0.895	56.774 **	0.864	0.018	0.992	1.248 **	0.301
	PgnC	0.984	17.641 ***	0.847	0.648 *	0.884	42.972 **	0.836	0.144	0.994	2.651 *	0.443
NLC-Lo-DSG	DSG	0.978	13.527	0.910	0.217	0.951	58.723	0.894	0.028	0.997	2.068	0.375
NLC-Lo-PgnC	PgnC	0.997	20.705	0.831	0.718	0.990	67.227	0.875	0.063	0.997	2.891	0.459
NLC-Lo-DSG-PgnC	DSG	0.983	15.080 **	0.823	0.328 **	0.958	58.704 **	0.893	0.028	0.999	1.903 *	0.358
	PgnC	0.997	20.467 **	0.917	0.401 **	0.968	65.339 ***	0.926	0.067	0.999	2.787 *	0.436

* *p* < 0.05; ** *p* < 0.005; *** *p* < 0.0005; *p* > 0.05. Data are expressed as mean ± SD, *n* = 3; NLC-To/Lo-DSG/PgnC vs. other groups.

## Data Availability

Not applicable.
